# Audit into post diagnostic support in newly diagnosed dementia patients

**DOI:** 10.1192/bjo.2021.878

**Published:** 2021-06-18

**Authors:** Madhumanti Mitra, Raghupathy Paranthaman

**Affiliations:** Greater Manchester Mental Health NHS Foundation Trust

## Abstract

**Aims:**

This audit aims to identify whether newly diagnosed dementia patients are offered post diagnostic support and potential factors influencing patient choice.

**Background:**

A diagnosis of dementia can be life changing and hence post-diagnostic support for dementia is key. Multiple guidelines suggest that post diagnostic support need to be offered to all patients diagnosed with dementia. The Department of Health and Social Care and other national/ local guidelines suggest that post diagnostic support is offered to all patients diagnosed with dementia.

**Method:**

Data were collected for 40 patients diagnosed with dementia. Using random number generator, patient group was selected from pool of patients diagnosed with dementia between July’ 2017 - December’ 2017. Data included whether they had been offered support during the initial appointment and what post-diagnostic support was offered. Demographic details obtained to identify patterns of support accessed by patients.

**Result:**

All patients were offered post-diagnostic support. Diagnosis was discussed in appointment in about 93% of patients. Medication was discussed in 82% patients. Driving was discussed in only 64% patients and LPA was discussed in only 63% patients. When given choice between Post diagnostic support group (PDSG) and Dementia adviser (DA), slightly more women tend to choose PDSG group. The only 2 ethnic minority patients chose DA. 21% more patients opted for PDSG group when they had a carer.

**Conclusion:**

The positive is that some post-diagnostic support is offered to all patients. Although discussion of diagnosis with patients was done well, discussion of medication, driving and LPA can be improved upon. Ethnicity and family structure/ carer may have a bearing on patient choice of post-diagnostic support.

